# Effects of Annealing Temperature on Interfacial Structure and Thermal Conductivity of Hot-Pressed Copper/Cr-Coated Diamond Composites

**DOI:** 10.3390/ma19081534

**Published:** 2026-04-11

**Authors:** Yajing Liu, Xiaohong Chen, Yong Liu, Wei Tian, Fanfan Zhou, Honglei Zhou, Yicheng Wang

**Affiliations:** 1 College of Science, University of Shanghai for Science and Technology, Shanghai 200093, China; liuyajingusst@163.com; 2 School of Materials Science and Engineering, University of Shanghai for Science and Technology, Shanghai 200093, China; cxh992@163.com (X.C.); liuyongusst@163.com (Y.L.);; 3 Co-Innovation Center for Energy Therapy of Tumors, Shanghai 200093, China

**Keywords:** diamond/copper composites, magnetron sputtering, carbides, interface design, thermal conductivity, interfacial thermal conductivity

## Abstract

Efficient heat dissipation is crucial for semiconductor devices; however, conventional thermal management materials often cannot meet practical demands because of inadequate thermal conductivity and mismatched coefficients of thermal expansion with semiconductor materials. In this study, we develop a synergistic process integrating magnetron sputtering and annealing to fabricate a composition-controllable Cr/Cr_3_C_2_ composite interlayer on diamond surfaces. By regulating the annealing temperature from 700 to 1100 °C, three key parameters of the Cr/Cr_3_C_2_ composite interlayer can be tailored: the thickness varies from ~200 to 800 nm, the Cr/Cr_3_C_2_ fraction is adjustable, and the surface roughness ranges from 33.3 to 61.6 nm. In the current research, the sample that was annealed at 900 °C for 2 h exhibited the highest coating uniformity, with carbide coverage exceeding 98% and no discernible porosity. This optimized annealing process produces an interlayer with robust coverage, moderate thickness (~300 nm), and low surface roughness (Ra = 33.3 nm), thereby markedly enhancing interfacial bonding and thermal-transport performance. The resulting composite achieves a maximum thermal conductivity of 605.27 W·m^−1^·K^−1^, corresponding to 211% of the experimentally measured value for the uncoated sample. Analyses combining the diffusion mismatch model and experimentation indicate that the enhancement originates from improved phonon spectral matching and increased interfacial adhesion energy. This work provides processing guidance for precise interface engineering in high-thermal-conductivity diamond/copper composites.

## 1. Introduction

Device scaling continues to push microprocessors, diodes, and laser diodes toward higher integration, but the accompanying increase in volumetric heat generation has become a critical constraint on reliability and performance [1]. Reported power densities in state-of-the-art transistors and chips can reach approximately 150 W/mm^2^, so insufficient heat removal may lead to temperature rise, accelerated degradation, and premature failure [2,3].

Conventional thermal management materials, including copper, aluminum, silicon, and silicon carbide, have inherent limitations. In many cases, their thermal conductivity is not sufficient, and their coefficient of thermal expansion (CTE) differs markedly from that of semiconductor materials (typical CTE: 2.4–7.2 × 10^−6^ K^−1^) [4,5]. Copper, for example, reaches a thermal conductivity of 386 W·m^−1^·K^−1^ at room temperature, yet its CTE (17 × 10^−6^ K^−1^) remains incompatible with semiconductor materials. Diamond is, therefore, regarded as an attractive candidate because it combines exceptionally high thermal conductivity (1500–2200 W·m^−1^·K^−1^) with a low CTE (1.2–4.5 × 10^−6^ K^−1^) [6]. However, its high cost, the complexity of fabrication, and CTE differences with electronic components limit its direct use in electronic devices.

Copper continues to draw scientific attention, owing to its favorable thermal performance, good machinability, and compatibility in practical processing. Accordingly, copper matrix composites reinforced with diamond particles have been widely investigated [7,8,9,10]. Despite these advantages, Cu/diamond composites still encounter difficulties in lowering thermal resistance at the interface. Poor wettability between copper and diamond hinders the formation of robust chemical bonding. The large Debye temperature disparity between copper (325 K) and diamond (2200 K) further aggravates phonon impedance and leads to strong phonon scattering at the interface [11,12]. Theoretical studies, including molecular dynamics simulations, suggest that introducing an interlayer with intermediate phononic characteristics can substantially enhance heat transfer [13,14,15]. The main approaches include matrix alloying with elements such as B or Zr [16,17,18], and metallizing diamond surfaces using carbide-forming elements such as Ti, Cr, Mo, and W [19,20,21,22,23,24,25]. Among these, surface metallization is often preferred because it offers a broader choice of modifying elements, enables precise control of coating thickness, and reduces adverse effects on the thermal conductivity of the copper matrix [24,26]. In contrast, alloying strategies typically decrease the intrinsic thermal conductivity of copper [27,28]. Well-established metallization techniques include salt bath plating, magnetron sputtering, and vacuum evaporation, among others [25,29,30,31]. Magnetron sputtering was selected in this study for its advantages in terms of producing uniform, dense coatings with precise thickness control down to nanometers [24,29].

Cr has been identified as a particularly effective interlayer element for several key reasons. First, its strong chemical affinity for carbon drives the formation of a stable Cr_3_C_2_ phase (∆G < 0), enabling durable chemical bonding at the interface [32]. Second, its Debye temperature lies between those of copper and diamond, allowing it to act as a bridge to reduce phonon spectrum mismatch [33,34,35,36]. Furthermore, its CTE (~4–6 × 10^−6^ K^−1^) falls between those of the two constituents, which helps to minimize thermally induced stress [29]. Wang et al. [31] recently used a molten salt method to deposit a chromium carbide layer of about 180 nm on the diamond surface, yielding a composite TC of 431 W·m^−1^·K^−1^. However, their study conducted limited microstructural analysis of the carbide layer and did not correlate the carbide ratio with TC. The interfacial thermal conductance between Cu and diamond is mainly dominated by two competing mechanisms. On the one hand, Cr atoms transform into Cr_3_C_2_ at high temperatures, and the Debye velocity ratio of Cr_3_C_2_ to diamond is greater than that of Cr to diamond. Generally, the interfacial thermal conductivity is proportional to the Debye sound velocity ratio of the metal film and the non-metal substrate; that is, the smaller the acoustic difference on both sides of the interface, the higher the interfacial thermal conductivity [37,38]. Therefore, the transformation of Cr into Cr_3_C_2_ will effectively enhance the interfacial thermal conductivity of the composite material. On the other hand, the complete transformation of Group VIB metals into carbides will actually reduce the interfacial thermal conductivity of the Cu/interlayer/diamond structure. For example, the complete transformation of Mo into Mo_2_C and the complete transformation of Cr into Cr_3_C_2_ will both reduce the interfacial thermal conductivity of the multilayer composite structure [19,39]. Clearly, the phase composition of the interface directly affects the interfacial thermal conductivity and, thus, the thermal conductivity of the composite material. To address these gaps, this study innovatively focuses on controlling the Cr/Cr_3_C_2_ ratio through annealing temperature, providing detailed microstructural analysis and theoretical modeling to correlate carbide fraction, thickness, and anisotropy with TC, offering a novel framework for interface design.

In this study, controllable Cr/Cr_3_C_2_ interlayers were fabricated on diamond surfaces through a magnetron sputtering and annealing process, with the annealing temperature set at 700 to 1100 °C. The thermal properties of the resulting composites were evaluated via laser flash analysis and infrared thermal imaging. The diffusion mismatch model (DMM) calculations were then combined with experiments to elucidate the underlying enhancement mechanisms, thereby establishing a direct link between the engineered interlayer structure and the composite thermal conductivity.

## 2. Materials and Methods

### 2.1. Materials

MBD6-type diamond particles (60–70 mesh) were obtained from Huanghe Whirlwind Co., (Xuchang, HenanChina). High-purity (99.99%) chromium and copper targets (Ø470 mm × 145 mm× 8 mm) backed by high-thermal-conductivity copper plates (Ø500 mm × 175 mm × 10 mm) were used for magnetron sputtering. The plasma gas was high-purity argon (Ar, 99.99%). For the electroless copper plating process, CuSO_4_·5H_2_O (15 g/L) and HCHO (37 wt.%) were used as the Cu source and reducing agent. NaOH, Na_2_EDTA·2H_2_O, and C_10_H_8_N_2_ were used as the pH adjuster, complexing agent, and stabilizer, respectively. Copper powders with two particle sizes (70 µm and 20 µm) were mixed in a 2:1 mass ratio for the composite matrix.

### 2.2. Experimental Procedures

Diamond particles underwent ultrasonic cleaning in acetone and ethanol (30 min each), followed by rinsing to a neutral pH. Chromium layers were deposited using a Teer magnetron sputtering system under an Ar plasma atmosphere, at a chamber vacuum of 1 × 10^−3^ Pa. Deposition conditions included a Cr sputtering power of 450 W, substrate bias voltage of 70 V, dual-target configuration, and substrate rotation at 4 revolutions per minute. The process lasted 10 min, producing uniform and dense coatings. Chromium-coated diamond particles were annealed in a tube furnace under an Ar/H_2_ flow to limit oxidation. The furnace was ramped up at 10 °C/min to 700–1100 °C, maintained for 2 h, and allowed to cool to room temperature in the furnace. Chromium carbide was formed as the primary interfacial product.

Annealed particles were subjected to an electroless copper plating process to form a dual-layer structure. The plating bath pH was maintained between 13 and 14 using NaOH, with 200 mL of Na_2_EDTA·2H_2_O (14 g/L) being added to prevent copper ion hydrolysis and with 0.004 g C_10_H_8_N_2_ acting as a stabilizer. The plated diamonds were mixed with copper powder (70 μm: 20 μm = 2:1 mass ratio) in ethanol for 3 h, maintaining a diamond volume fraction of 50%.

**Figure 1:**
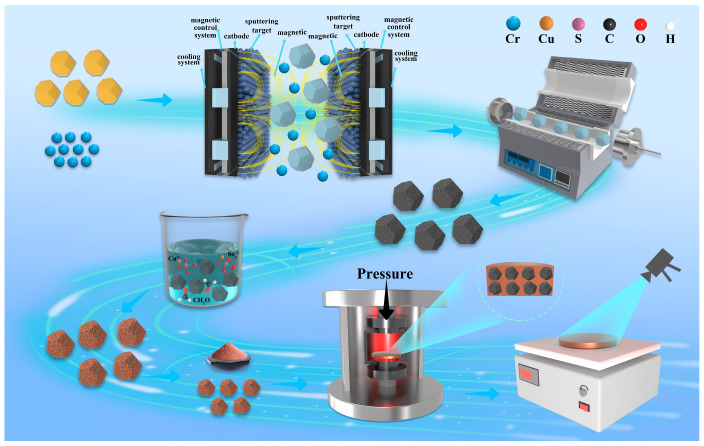
Schematic of the diamond and copper composite preparation process.

The mixed powders were loaded into graphite dies lined with graphite paper to prevent adhesion. Sintering was performed using a hot-pressing system with combined induction/electrical heating under a vacuum environment. The temperature was raised to 850 °C at a rate of 10 °C/min under an applied pressure of 25 kN, followed by a holding time of 30 min. The final sintered samples were cylindrical, with dimensions of Ø12.7 mm × 3 mm. The synthesis process is illustrated in Figure 1. For clarity, the samples are designated as follows: uncoated diamond (L_A_) samples and annealed specimens at 700 °C (L_B_), 900 °C (L_C_), and 1100 °C (L_D_) for 2 h.

### 2.3. Sample Characterization

The crystalline structure was characterized using X-ray diffraction (XRD, D8 Bruker, Karlsruhe, Germany) in a scanning range from 20° to 80°, at a scanning rate of 5°/min. Surface morphology examination was conducted using scanning electron microscopy (SEM, JSM–IT500HR, JEOL, Tokyo, Japan). X-ray photoelectron spectroscopy (XPS, Shimadzu AXIS Supra^+^, Kyoto, Japan) was employed to analyze the chemical states and elemental valence distribution of the chromium coatings and copper plating. Surface roughness measurements were performed with an atomic force microscope (AFM, Dimension ICON Bruker, Karlsruhe, Germany) operated in tapping mode, using a scan area of 5 μm × 5 μm and a scan frequency of 0.9 Hz. A HIKMICRO infrared thermal imager(Wuhan Lanmi Trading Co., Ltd., Wuhan, China) was used to collect infrared images. Thermal diffusivity (α) was measured at 25 °C using a laser-flash analyzer (LFA467, Netzsch, Selb, Germany) with an accuracy of ±3%. Density (ρ) values were obtained through the Archimedes water immersion method. The specific heat capacity ($C_{p}$) of the composites was calculated by applying the rule of mixtures, a well-established methodology for Cu/diamond composites [40]. Thermal conductivity (λ) was subsequently derived using the relationship $\lambda =\alpha \cdot \rho \cdot C_{p}$ (with measurements repeated three times for each sample).

## 3. Results and Discussion

### 3.1. Surface Morphology, Phase Composition, and Bonding States of the Coating Layer

**Figure 2:**
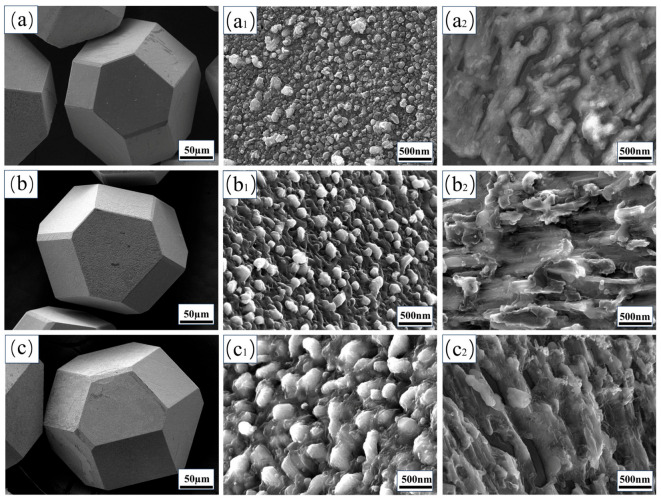
Surface morphology of Cr-coated diamond particles extracted from the corresponding Cu/diamond composites after sintering: (**a**–**a_2_**) L_B_, (**b**–**b_2_**) L_C_, (**c**–**c_2_**) L_D_, (**a_1_**–**c_1_**) the {100} plane, and (**a_2_**–**c_2_**) the {111} plane.

Figure 2 presents the surface morphology of Cr-coated diamond particles extracted from the corresponding Cu/diamond composites after sintering, demonstrating effective retention of their original polyhedral morphology following coating deposition. The coating coverage observed on diamond facets with different crystallographic orientations confirms that magnetron sputtering can produce uniform coatings and largely suppresses crack formation during subsequent annealing [24]. At lower annealing temperatures, the L_B_ series forms relatively thin carbide layers on the {100} and {111} diamond surfaces. These carbide layers consist of fine particles with a non-uniform distribution (Figure 2(a–a_2_)). Raising the annealing temperature from 700 to 1100 °C leads to clear changes in surface morphology. Grain size increases progressively with temperature, which is consistent with accelerated atomic diffusion and recrystallization at elevated temperatures. As shown in Figure 2b,c, the L_C_ and L_D_ coatings form a dense, continuous surface layer composed of aggregated chromium-containing particles.

Carbide growth mainly proceeds via two mechanisms: lateral spreading, dominated by the surface diffusion of carbon atoms, and vertical thickening, governed by slower bulk diffusion [41]. This accounts for the experimentally observed gradual coalescence of carbide islands and the time-dependent increase in surface coverage. A high carbide coverage is crucial for optimizing the heat-transport pathway. Therefore, an L_C_ coating with near-complete coverage and a moderate thickness was expected to yield the highest thermal conductivity in the final composite samples.

On the diamond {100} facet, a uniform carbide layer composed of fine-grained features formed, whereas the {111} facet exhibited larger, irregular plate-like carbide structures (Figure 2(a_1_–c_1_,a_2_–c_2_)). These observations are consistent with those reported by Wang et al., showing that ZrC preferentially nucleates at high-density pits on {100} facets and at low-density vertical steps on {111} facets [18], and they further corroborate a previous finding that such step features can form on {111} facets during vacuum annealing [42].

For the L_C_ samples, the carbide coverage on both facets exceeded 98%, forming an essentially complete and continuous layered structure. Surface carbon atoms on the {100} facet were mainly double-bonded, while those on the {111} facet were mainly triple-bonded. This difference in carbon bonding may account for the larger particle size on the {111} facet by altering the reaction kinetics [8].

The surface energy of diamonds is 9.4 J·m^−2^ for the {100} facet and 5.4 J·m^−2^ for the {111} facet [43], and this anisotropy in interfacial reaction activity is closely associated with this facet-dependent surface energy. The unique atomic arrangements of these facets and their associated physicochemical properties underpin their differentiated interactions with metals at elevated temperatures. Similar crystallography-dependent reaction behaviors have been documented in many metal-diamond systems, including Al/diamond composites, Cu alloys, and Fe-, Co-, and Ni-based alloys [37,44,45], indicating the generality of this phenomenon.

**Figure 3:**
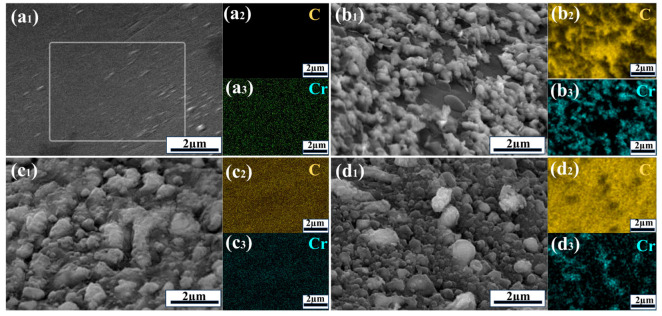
EDS analysis of Cr-coated diamond particles extracted from different Cu/diamond composites: (**a_1_**–**a_3_**) untreated, (**b_1_**–**b_3_**) L_B_, (**c_1_**–**c_3_**) L_C_, and (**d_1_**–**d_3_**) L_D_.

Figure 3 presents the EDS analysis of Cr-coated diamond particles extracted from different Cu/diamond composites. The EDS elemental analysis results show that as the annealing temperature increases, the Cr element content in the Cr/Cr_3_C_2_ coating layer gradually decreases, while the C element content gradually increases, indicating that the Cr coating gradually transforms into Cr_3_C_2_ carbide. This result reveals the phase evolution mechanism of the Cr/Cr_3_C_2_ coating layer during the annealing process. The EDS energy spectrum analysis of the unannealed sample shows that only the Cr element is detected on the surface of the diamond particles, while almost no C element is detected. This indicates that before annealing, the coating layer on the diamond surface is mainly composed of pure metal Cr, and there is only a simple physical combination between Cr and diamond, without obvious chemical bonding reactions. This is mainly because the reactivity of Cr at room temperature is low, and it is difficult for it to react chemically with the C atoms on the diamond surface; at room temperature, the atomic diffusion rate is extremely slow, and there is no diffusion driving force between the Cr atoms and C atoms, so no carbide can be formed. As the annealing temperature increases, the Cr coating gradually transforms into Cr_3_C_2_ carbide. Both the atomic diffusion mechanism and the chemical reaction mechanism at high temperatures dominate the mutual diffusion and bonding reactions between Cr atoms and C atoms on the diamond surface. The EDS energy spectrum analysis shows that the Cr element content gradually decreases and the C element content gradually increases with an increase in annealing temperature, indicating that the degree of chemical reaction between Cr and C increases with an increase in temperature. At high temperatures, the Cr_3_C_2_ grains will grow, and their morphology will change accordingly. The EDS energy spectrum analysis shows that the distribution of the C element in the Cr coating gradually changes from a locally uniform distribution to an overall uniform distribution, indicating that the Cr_3_C_2_ grains gradually grow and cover the entire diamond surface.

**Figure 4:**
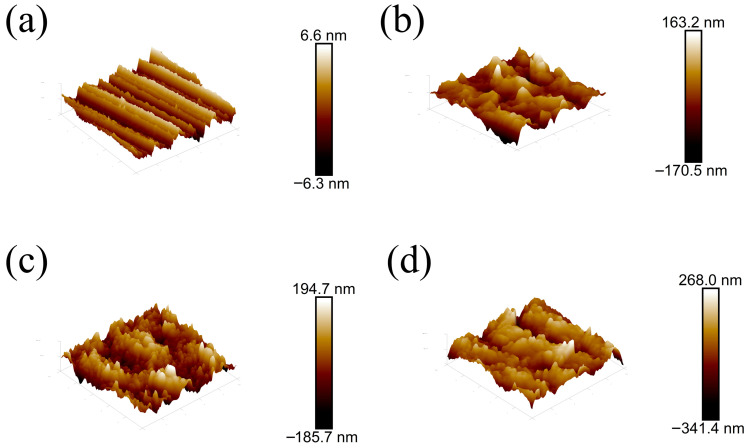
AFM images of the diamond surfaces coated with different Cr layers: (**a**) L_A_, (**b**) L_B_, (**c**) L_C_, and (**d**) L_D_.

Heat-treatment temperature governs the morphological characteristics of the surface coating on the samples; from the AFM observations (Figure 4), we found that coatings generally increased the roughness of originally smooth diamond grains, where the pristine diamond substrate exhibited an arithmetic mean roughness of 1.58 nm and the roughness after treatments at 700 °C, 900 °C, and 1100 °C was 45.6 nm, 33.3 nm, and 61.6 nm, respectively. Specifically, the 700 °C-treated L_B_ coating was rougher than the L_C_ sample due to incomplete chromium-to-carbide conversion and non-uniform morphology, whereas the 1100 °C-treated L_D_ coating had the highest level of roughness because coarse carbides formed at high temperature, which is consistent with the SEM images, and the 900 °C-treated L_C_ coating showed the optimal uniform and smooth morphology, with the lowest roughness among all samples.

Hopkins et al. [46] showed that the thickness of the intermixing region at material interfaces strongly affects thermal transport, implying that Cu/diamond composites should be engineered to maximize carbide coverage while keeping the carbide interlayer as thin as possible; moreover, prior work consistently reports an inverse relationship between surface roughness and thermal-transport efficiency [47,48], while studies by Duda et al. and Gundrum et al. [49,50] further suggest the low-temperature convergence of interfacial thermal conductivity because roughness preferentially scatters phonons with wavelengths smaller than the characteristic roughness scale, thereby causing electron transport at metal interfaces governed by disordered elastic scattering and leading to a more extensive disordered region between surface peaks and valleys at higher roughness [51]. Furthermore, multidimensional analysis indicates that the L_C_ coating provides the optimal combination of low roughness, suitable carbide thickness, and high carbide coverage, which explains its superior interfacial thermal conductivity and shows the importance of tailored interface engineering for advanced thermal management.

**Figure 5:**
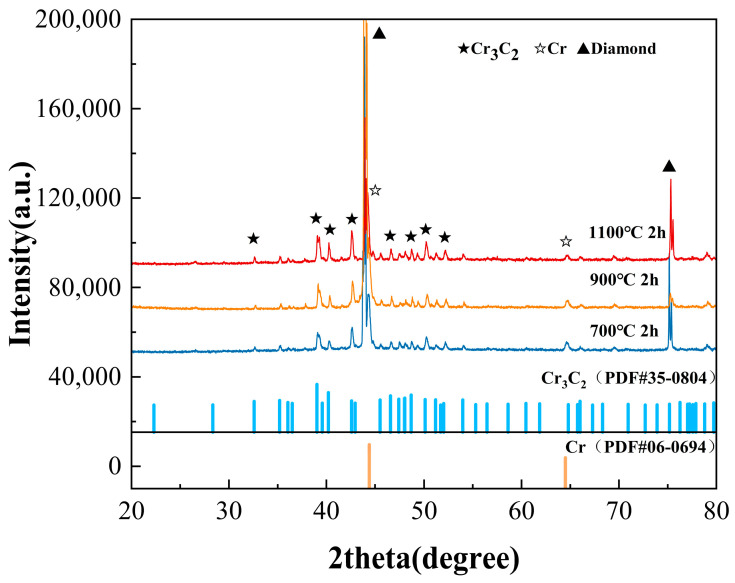
XRD pattern of Cr-coated diamond particles extracted from different Cu/diamond composites.

Figure 5 shows the XRD patterns of the Cr/diamond samples, which exhibit distinct diffraction peaks that are attributable to diamond (PDF#06-0675), metallic Cr (PDF#06-0694), and chromium carbide Cr_3_C_2_ (PDF#35-0804). Specifically, the Cr coating displays characteristic reflections from the (110) and (200) planes at 44.4° and 64.6°, respectively, whereas the Cr_3_C_2_ phase presents identifiable peaks at 39.0° (121), 40.2° (230), 42.5° (150), 50.2° (310), etc. Phase analysis confirms that, under different heat-treatment conditions, the film on the Cr/diamond samples is composed of Cr_3_C_2_ and Cr. After heat treatment at 700 °C for 2 h, weak Cr_3_C_2_ diffraction peaks appear, suggesting that chemical reactions have been initiated between Cr atoms in the metallic layer and carbon atoms on the diamond surface.

As shown in Figure 5, increasing the heat-treatment temperature progressively weakens the diffraction signal of metallic Cr while markedly enhancing the Cr_3_C_2_ carbide peaks. This trend indicates that during high-temperature annealing, the Cr coating gradually transforms from a metallic layer into a carbide-dominated structure. Elevated temperatures provide higher activation energy for interfacial reactions between Cr and carbon atoms, thereby accelerating the conversion of metallic Cr to Cr_3_C_2_ and supplying a greater driving force for grain growth [52]. The XRD results further suggest that carbide formation shifts the interfacial nature from physical vapor deposition contact to a chemically bonded connection. Such a phase evolution enhances interfacial wettability, thereby improving the overall performance of the composite. The phase fractions quantified using XRD are summarized in Table 1.

The texture coefficients of different crystal planes of Cr and Cr_3_C_2_ are summarized in Table 2. We systematically correlated the texture coefficients with the surface morphology and roughness features observed by SEM and deeply explored the influence of texture on the structure and properties of the Cr/Cr_3_C_2_ coating. As a function of crystalline size, the texture coefficient represents the texture of a specific plane, and its deviation from the standard sample indicates the preferential growth of that crystal plane. The thickness of the film has a direct impact on the texture of the grown material. Moreover, the structure and photoelectric properties of the material, as well as the performance and reliability of the fabricated devices, are significantly affected by the material texture [53]. The texture coefficient of each (hkl) plane is determined from the XRD spectrum, according to the following formula.


(1) $$TC_{\left(h_{i}k_{i}l_{i}\right)}=\frac{I_{\left(h_{i}k_{i}l_{i}\right)}}{Io_{\left(h_{i}k_{i}l_{i}\right)}}{\left\{\frac{1}{N}\sum_{i=1}^{N}\frac{I_{\left(h_{i}k_{i}l_{i}\right)}}{Io_{\left(h_{i}k_{i}l_{i}\right)}}\right\}}^{-1}$$

Here, $I_{\left(h_{i}k_{i}l_{i}\right)}$ is the intensity of the $\left(h_{i}k_{i}l_{i}\right)$ diffraction peak of the studied sample; $Io_{\left(h_{i}k_{i}l_{i}\right)}$ is the intensity of the $\left(h_{i}k_{i}l_{i}\right)$ plane of a completely random sample from the powder diffraction file (PDF) card; N is the number of diffraction times considered in the analysis. Table 2 shows the changes in texture coefficient values of several main peaks of Cr and Cr_3_C_2_. When the TC value is greater than 1, this indicates that the crystal plane has preferential orientation, and the larger the value, the higher the degree of preferential orientation; when the texture coefficient value is less than 1, this indicates that the orientation degree of the crystal plane tends to be randomly distributed [53]. As shown in Table 2, the (110) and (200) crystal planes of the Cr phase in the LB coating layer have obvious preferential orientation, while the texture of the Cr_3_C_2_ phase is weak (the texture coefficient of (230) is only 0.28); the SEM image shows that the surface of the L_B_ coating layer is composed of fine Cr grains and Cr_3_C_2_ grains that have just begun to nucleate, with a relatively low surface roughness (Ra = 45.6 nm). The (200) crystal plane of the Cr phase in the L_C_ coating layer has a certain degree of preferential orientation (texture coefficient = 1.22), while that of (110) is only 0.95, and the (150) crystal plane of the Cr_3_C_2_ phase has obvious preferential orientation (texture coefficient = 2.10), which can confirm that the Cr phase in the L_C_ coating layer has been largely transformed into the Cr_3_C_2_ phase; the SEM image shows that the surface of the coating layer is mainly composed of uniformly grown Cr_3_C_2_ grains in situ, with the (150) crystal plane of the Cr_3_C_2_ phase having a strong growth advantage, forming a medium-sized grain structure, and its surface roughness is the lowest (Ra = 33.3 nm). The (150) crystal plane of the Cr_3_C_2_ phase in the L_D_ coating layer has obvious preferential orientation (texture coefficient = 2.26), while the texture degree of the (200) crystal plane of the Cr phase is the weakest among the three; the SEM image shows that the surface of the coating layer is composed of coarse nucleated Cr_3_C_2_ grains with a relatively high surface roughness (Ra = 61.6 nm), which is due to the abnormal growth of Cr_3_C_2_ grains during high-temperature annealing, and the (200) crystal plane of the Cr phase acts as a nucleus to promote the growth of Cr_3_C_2_ grains forming a coarse grain structure, thereby resulting in a relatively high surface roughness. The results show that the Cr film deposited at room temperature has obvious (200) preferential orientation, and that as the annealing temperature increases, the Cr (200) texture gradually weakens, while the Cr_3_C_2_ (230) and (150) textures gradually strengthen.

**Figure 6:**
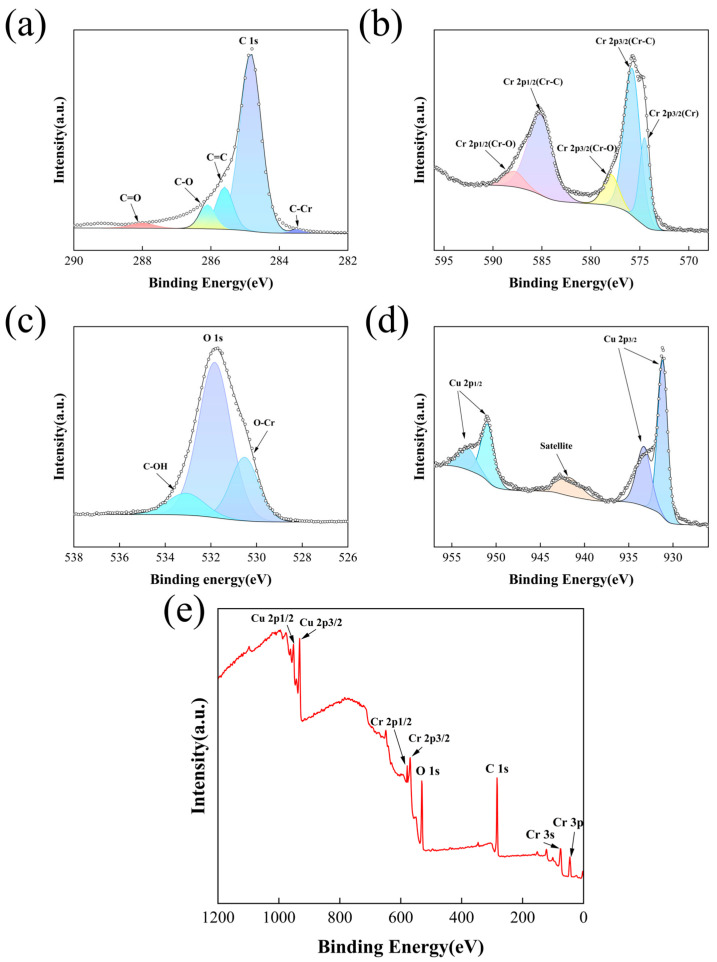
XPS core-level spectra of the diamond particles (L_C_, 900 °C for 2 h) with a double coating of chromium and copper: (**a**) C 1s, (**b**) Cr 2p, (**c**) O 1s, (**d**) Cu 2p, and (**e**) wide-spectrum XPS.

XPS was employed to characterize the surface composition and chemical bonding states of extracted chromium- and copper-plated diamond particles (L_C_ sample). As shown in Figure 6a–e, photoelectron spectra were collected for the C 1s (282–290 eV), Cr 2p (570–595 eV), O 1s (526–538 eV), and Cu 2p (926–957 eV) regions. Peak deconvolution of the C 1s spectrum resolves into five components at binding energies of 283.5, 284.8, 285.6, 286.1, and 288.1 eV, which are assigned to the C-Cr, C-C (sp^3^), C=C (sp^2^), C-O, and C=O species, respectively [31,54,55]. The dominant carbon signal in the survey spectrum originates from the diamond substrate. In the Cr 2p region, two characteristic doublets at 575.8 eV and 585.4 eV are consistent with the C-Cr bonding state in chromium carbide (Cr_3_C_2_), indicating the presence of a carbide interlayer on the diamond surface. Additional peaks at 577.9 eV and 587.9 eV correspond to Cr-O bonds, suggesting surface oxidation induced by air exposure. The O 1s spectrum further confirms the presence of metal–oxygen and carbon–oxygen bonding, supporting the formation of a surface oxide layer. In the Cu 2p region, the peaks at 932.3 eV and 952.2 eV are assigned to metallic Cu^0^, whereas those at 934.1 eV and 954.1 eV correspond to Cu^2+^ [56], which may exist as CuO. The detection of copper oxides suggests that the reduction that occurs during electroless plating is not complete and that residual oxidation can occur upon exposure to air. The XPS spectra indicate the presence of Cr_3_C_2_, together with Cu and surface oxides, supporting a chemically heterogeneous interface that affects interfacial bonding and the thermal response of the composite.

### 3.2. Thermal Conductivity and Thermophysical Performance of the Composites

**Figure 7:**
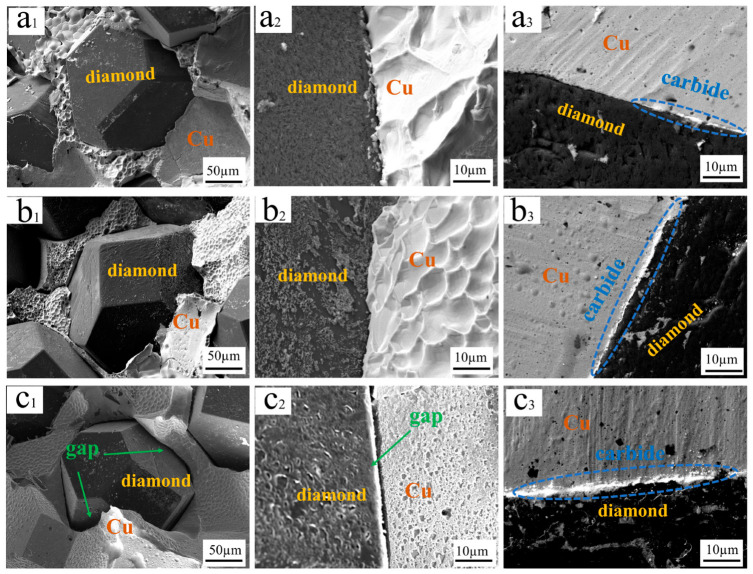
Interfacial bonding states of composites reinforced by Cr-coated diamond particles annealed at different temperatures: (**a_1_**–**a_3_**) L_B_, (**b_1_**–**b_3_**) L_C_, and (**c_1_**–**c_3_**) L_D_.

Direct contact between diamond particles and copper provides weak interfacial adhesion, which is evidenced by pores and microcracks and is associated with higher interfacial thermal resistance [57]. Introducing a chromium interlayer improves interfacial continuity. The resulting Cr_3_C_2_ and Cr transition layer separates the diamond particles from the copper and reduces crack formation and pore size (Figure 7). Fracture surface analysis (Figure 7(a_1_–c_1_,a_2_–c_2_)) shows that the coating bridges the two phases [58], forming chemical bonds with the diamond particles while improving wettability with the copper matrix.

Figure 7 shows the clear evolution of the interface with heat treatment and temperature. The spatial relationship between Cu and diamond particles is evident in the labeled images, with the Cu matrix surrounding the diamond particles and with carbide interlayers bridging them. At 700 °C (L_B_), the interfacial layer is discontinuous, and residual porosity remains (Figure 7(a_1_–a_3_)). At 900 °C (L_C_), the interface is tightly bonded, and a continuous chromium carbide interlayer is obtained (Figure 7(b_1_–b_3_)). When the temperature is increased to 1100 °C (L_D_), the interlayer thickens, yet the carbide grains coarsen, and gaps appear at the interface (Figure 7(c_1_–c_3_)). The amount of copper adhering to the diamond surface follows the same trend, being highest for L_C_, partial for L_B_, and minimal for L_D_, where the diamond surface is largely exposed. Such behavior likely reflects accelerated carbide grain growth at elevated temperature, which increases interlayer thickness but disrupts interfacial continuity [8,22].

High-magnification cross-sectional images (Figure 7(a_3_–c_3_)) further reveal interfacial details. The L_C_ coating is characterized by an appropriate thickness and a uniform microstructure, whereas the L_B_ coating appears relatively thin and the L_D_ coating has become excessively thick, due to grain coarsening. Under high-temperature treatment, the presence of a graphite phase further degrades the thermal conductivity of the composite material [59]. Therefore, the composite sample annealed at 900 °C exhibits the best thermal performance, benefiting from an outer copper coating that promotes the formation of a network structure during hot pressing. Electroless copper plating enables uniform deposition via chromium-based nucleation sites, thereby shifting the sintering process from diamond–copper diffusion to copper–copper diffusion and consequently enhancing low-temperature densification and interfacial bonding strength.

**Figure 8:**
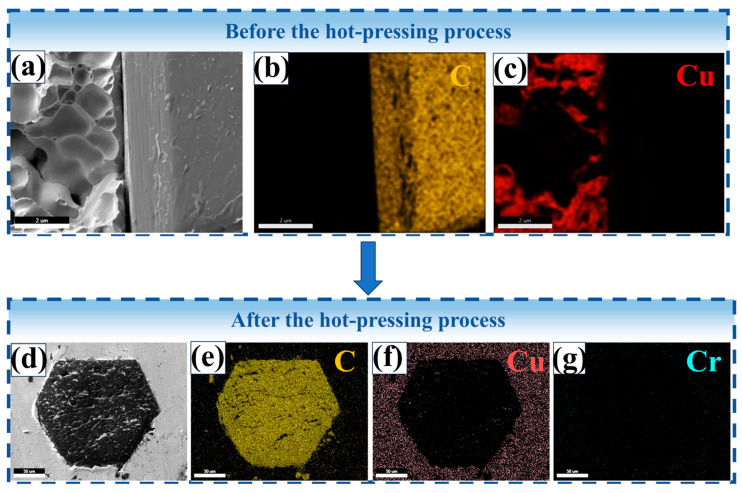
EDS energy spectrum analysis of samples before and after the hot-pressing process: (**a**–**c**) before the hot-pressing process; (**d**–**g**) after the hot-pressing process.

We conducted EDS energy spectrum elemental analysis of the interface bonding state of the composite material before and after the hot-pressing process, as shown in Figure 8. The results indicated that the interface bonding state between the copper and diamond particles was poor before the hot-pressing process, but after the hot-pressing process, a tight metallurgical bond was formed between the copper matrix and the diamond particles, and obvious carbon and chromium element infiltration was observed in the copper matrix. This result reveals the microstructure evolution mechanism of the copper–diamond interface during the hot-pressing process. Before the hot-pressing process, the copper matrix and diamond particles were in a physical mixture state. EDS energy spectrum analysis showed that there was almost no copper element attached to the surface of the diamond particles, and no carbon element was detected on the surface of the copper matrix. This indicates that only mechanical contact existed between the copper and diamond particles before sintering, and the interface bonding force mainly originates from poor wettability between the copper and diamond particles, with extremely low interface bonding strength. This phenomenon was mainly due to the chemical inertness of the diamond surface and poor wettability with the copper matrix. After the hot-pressing process, EDS energy spectrum analysis showed that a layer of copper matrix fragments was attached to the surface of the diamond particles, and obvious carbon and chromium element infiltration was observed in the copper matrix, indicating that a tight metallurgical bond was formed between the copper and diamond particles. The significant improvement in interface bonding strength was mainly attributed to the following reasons: during the hot-pressing process, the combined effect of high temperature and pressure caused the molten copper matrix to undergo fluidity deformation, and copper atoms filled the microcracks and defects on the surface of the diamond particles, forming an interlocking structure. Additionally, at high temperatures, copper atoms and carbon atoms on the surface of the diamond particles underwent mutual diffusion, forming a copper–carbon solid solution transition layer. EDS energy spectrum analysis showed that the content of carbon elements in the copper matrix gradually decreased from the surface to the interior, indicating that carbon elements diffused from the surface of the diamond particles into the copper matrix. At the same time, copper elements also diffused to a certain extent onto the surface of the diamond particles, forming a thin copper–carbon solid solution layer on the surface of the diamond particles. Finally, and most importantly, at high temperatures, the chromium elements reacted chemically with the carbon atoms on the surface of the diamond particles to form Cr_3_C_2_. The Cr and Cr_3_C_2_ interlayer effectively bridged the phonon spectra characteristics of copper and diamond; this enhancement of thermal conductivity originates from improved phonon spectral matching and increased interfacial adhesion energy. EDS energy spectrum analysis showed that obvious chromium element infiltration was observed in the copper matrix, indicating that chromium elements diffused into the interior of the copper matrix and participated in the interface chemical reaction.

**Figure 9:**
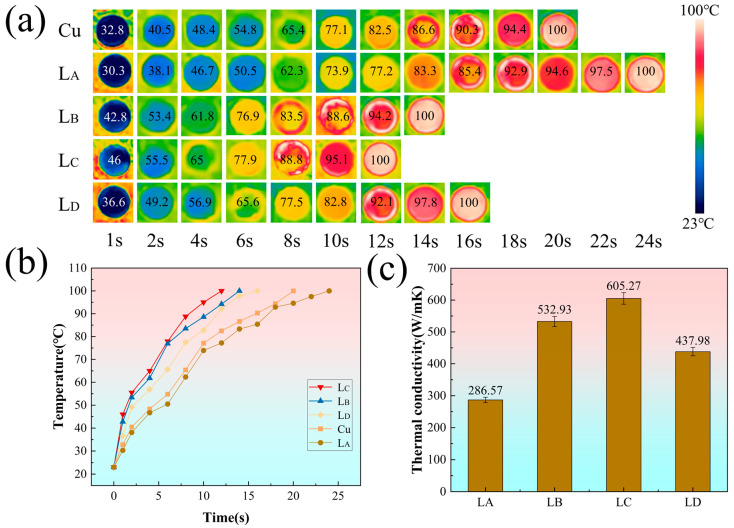
(**a**) The infrared thermal images, (**b**) the temperature-time curves of diamond/Cu composites with different metallic layers, and (**c**) the thermal conductivity values of diamond/Cu composites with different metallic layers.

The heat-transfer capability of the as-fabricated diamond/copper composites was evaluated using a customized real-time temperature monitoring system. Samples with identical dimensions were placed on a microelectronic heating platform maintained at 100 °C, and the evolution of their surface temperature was recorded by infrared thermography. Figure 9a presents thermal images acquired at consecutive time points (1, 2, 4, 6, 8, 10, 12, 14, 16, 18, 20, 22, and 24 s), showing that all composite samples exhibit a superior thermal response compared with pure copper. The temperature–time curves in Figure 9b indicate that the composite incorporating the L_C_ interlayer achieves higher heat-transfer efficiency. The L_C_-modified composite reaches the target temperature of 100 °C within 12 s, with a heating rate faster than that of the unmodified composite (by 12 s) and pure copper (by 8 s).

The thermal performance data of the as-fabricated Cu/diamond composites are summarized in Table 3. The uncoated diamond/copper composite exhibits a relatively low thermal conductivity of ~286 W·m^−1^·K^−1^, mainly due to poor interfacial bonding arising from the intrinsic non-wettability between diamond and copper. After applying a chromium coating on the diamond particle surfaces, the interfacial characteristics are markedly improved, leading to a substantial enhancement in the thermal performance of the composites. As shown in Figure 9c, the maximum thermal conductivity of the Cr-modified Cu/diamond composite reaches 605.27 W·m^−1^·K^−1^. This value is competitive compared with recent results in the literature, e.g., 431 W·m^−1^·K^−1^ from molten salt Cr-carbide [31], 469 W·m^−1^·K^−1^ with V-based coatings [60], and 550 W·m^−1^·K^−1^ with W-based coatings [7], demonstrating advantages in terms of process simplicity and cost-effectiveness while achieving high TC through optimized annealing. More importantly, this study establishes a clear correlation between interlayer composition, thickness, roughness, and thermal conductance—a systematic insight that was rarely provided in prior works.

### 3.3. Interface Characterization and Enhancement Mechanisms for Thermal Conductivity

Interfacial thermal conductivity plays a crucial role in determining the overall thermal conductivity of composite systems [11]. To evaluate the thermal conductivity of the diamond/Cu composites, the Hasselman–Johnson (H–J) model deduced in the Maxwell mean field (MMF) scheme is used [4,61]. The thermal conductivity λ of the composite can be described as follows:

(2) $$\lambda =\lambda_{m}\left[\frac{2\left(\frac{\lambda_{d}}{\lambda_{m}}-\frac{\lambda_{d}}{ah}-1\right)V_{d}+\frac{\lambda_{d}}{\lambda_{m}}+\frac{2\lambda_{d}}{ah}+2}{\left(1-\frac{\lambda_{d}}{\lambda_{m}}+\frac{\lambda_{d}}{ah}\right)V_{d}+\frac{\lambda_{d}}{\lambda_{m}}+\frac{2\lambda_{d}}{ah}+2}\right]$$

where λ, *$\lambda_{m}$*, *$\lambda_{d}$* are the thermal conductivities of the composite, matrix, and diamond, respectively, W⋅m^−1^ K^−1^; $V_{d}$ is the volume fraction of the reinforcement; a is the radius of spherical reinforcement, m; and h is the interfacial thermal conductance, W·m^−2^ K^−1^.

Phonon-mediated heat transfer across dissimilar materials is often analyzed using the diffusion mismatch model (DMM) [62], which captures the role of interfacial thermal resistance in heterogeneous structures. DMM represents the phonon impedance of the material, which is an indicator of phonon transmittance and can be calculated from the material density and the Debye phonon velocity. The DMM assumes diffuse phonon scattering at the interface, so the transmission probability depends primarily on the phonon density of the states of the adjoining materials. Accordingly, the interfacial thermal conductance predicted by the DMM is written as [63]:


(3) $$h=\frac{1}{4}\frac{\rho_{1}c_{1}\nu_{1}^{3}}{\nu_{1}^{2}+\nu_{2}^{2}}$$

The effect of the carbide interlayer on interfacial heat transport in copper/diamond composites was assessed with the DMM. The model yields the interfacial thermal conductance value, and the interfacial thermal resistance value was calculated from its inverse. The input parameters are listed in Table 4 [34,60].

Reducing the interfacial thermal resistance between copper and diamond is crucial for improving the thermal conductivity of diamond/copper composites [12]. The thermal conductivity between Cu and diamond in a Cu/intermediate/diamond structure can be represented by the concept of series thermal resistance:

(4) $$\frac{1}{h_{Cu/diamond}}=\frac{1}{h_{Cu/interlayer}}+\frac{d_{interlayer}}{K_{interlayer}}+\frac{1}{h_{interlayer/diamond}}$$

where d and K are the thickness and thermal conductivity of the intermediate layer, respectively. The interfacial thermal resistance between Cu and diamond includes five components: the interfacial thermal resistance of diamond/Cr_3_C_2_, the intrinsic thermal resistance of Cr_3_C_2_, the interfacial thermal resistance of Cr_3_C_2_/Cr, the intrinsic thermal resistance of Cr, and the interfacial thermal resistance of Cu/Cr.


(5) $$\frac{1}{h_{Cu/diamond}}=\frac{1}{h_{diamond/{Cr}_{3}C_{2}}}+\frac{d_{{Cr}_{3}C_{2}}}{K_{{Cr}_{3}C_{2}}}+\frac{1}{h_{{Cr}_{3}C_{2}/Cr}}+\frac{d_{Cr}}{K_{Cr}}+\frac{1}{h_{Cu/Cr}}$$

Based on the thermal conductivity of Cr_3_C_2_ and Cr, as given in Table 4, the interfacial thermal conductivity and interfacial thermal resistance values for different carbide phase compositions calculated by the DMM are shown in Table 5.

The interfacial thermal conductance values calculated using the H–J model (Table 6), together with the variation in the overall thermal conductivity of the composites, indicate that the interfacial thermal conductance (h) is the primary factor governing the dependence of composite thermal conductivity on heat-treatment temperature. These findings further emphasize the critical correlation between interfacial heat transport and the interfacial bonding state. During magnetron sputtering deposition, high-energy metal species (15–50 eV) bombard and are then deposited onto the diamond substrate, yielding an interfacial bonding strength exceeding 100 mJ·m^−2^. When the interfacial bonding strength surpasses this threshold, any enhancement in the thermal conductivity of Cu/diamond composites mainly arises from improved acoustic-impedance matching between the constituent materials [64]. In this study, the interfacial thermal conductance of all Cr-coated composites is higher than that of the unmodified Cu/diamond system.

Theoretical analysis confirms that when the acoustic impedance satisfies the relationship Z_Cu_ < Z_interlayer_ < Z_diamond_, the phonon transmission coefficient of an interlayer-structured interface is higher than that of an interface without an interlayer. This indicates that a rationally designed interlayer can effectively enhance interfacial heat-transport performance. As shown in Table 5, the monotonically increasing acoustic-impedance gradient from Cu to Cr/Cr_3_C_2_ and then to diamond enables the Cr/Cr_3_C_2_ transition layer to effectively bridge the phonon-vibration mismatch between Cu and diamond.

The interfacial thermal conductance between Cu and diamond particles is jointly governed by the thermophysical properties of the constituent phases within the Cu/Cr/Cr_3_C_2_/diamond multilayer structure. This property exhibits a non-monotonic dependence on heat-treatment temperature, first increasing and then decreasing, and is mainly dominated by two competing mechanisms. On the one hand, the Debye-velocity ratio between the interlayer and the diamond substrate strongly affects phonon transmission efficiency. Compared with the Cr–diamond system, the Cr_3_C_2_–diamond system has a higher Debye-velocity ratio and thus achieves better acoustic-impedance matching [37]. This explains, as shown in Table 5, the substantial increase in interfacial thermal conductance for the diamond/Cr_3_C_2_ interface (689.72 MW·m^−2^·K^−1^) relative to the diamond/Cr interface (399.51 MW·m^−2^·K^−1^). On the other hand, the heat-transport characteristics at the metal–interlayer interface differ markedly. As indicated by the data in Table 5, the interfacial thermal conductance of Cu/Cr (645.77 MW·m^−2^·K^−1^) is higher than that of Cu/Cr_3_C_2_ (461.07 MW·m^−2^·K^−1^). In addition, the intrinsic thermal conductivity of Cr_3_C_2_ is lower than that of Cr, Cu, or diamond. Therefore, once the carbide layer thickness exceeds the optimal value, excessive thickening will diminish the overall heat-transport performance.

In this study, a magnetron sputtering–annealing synergistic process was employed to precisely control the composition and thickness of the interfacial layer, thereby optimizing the Cu–diamond interfacial structure to fabricate high-thermal-conductivity composites. Theoretical analysis indicates that although the intrinsic thermal conductivity of commonly used carbide interlayers is typically lower than 25 W·m^−1^·K^−1^ and the interfacial thermal resistance introduced by the interlayer increases with its thickness [65], an overly thin interlayer is also insufficient to effectively improve interfacial bonding [66]. Therefore, in the Cu/Cr/Cr_3_C_2_/diamond system, the optimal interlayer thickness that balances interfacial thermal resistance and bonding strength must be determined experimentally. The experimental results indicate that a mixed Cr/Cr_3_C_2_ interlayer with a moderate degree of carburization gives the most favorable performance. In comparison, the L_C_ interlayer is continuous and uniform and offers a better balance among thickness, roughness, and bonding strength. With controlled carburization of the chromium layer, the highest thermal conductivity is obtained when the interlayer contains 77.1% Cr_3_C_2_ and 23.9% Cr, reaching 605.27 W·m^−1^·K^−1^. This composition strengthens interfacial bonding while maintaining a favorable carbide morphology and limiting interfacial thermal resistance, leading to improved heat transport performance in the composite.

## 4. Conclusions

This work establishes a synergistic magnetron-sputtering and annealing route that enables precise, temperature-controlled fabrication of a composition-tunable Cr/Cr_3_C_2_ composite interlayer on diamond surfaces. By systematically varying the annealing temperature (700–1100 °C), three critical interlayer parameters—thickness (about 200–800 nm), Cr/Cr_3_C_2_ phase ratio, and surface roughness (33.3–61.6 nm)—can be tailored, thereby overcoming the limitations of conventional metallization methods that offer limited compositional control.

This interfacial engineering produces dramatic improvements in thermal conductivity (TC) relative to the uncoated Cu/diamond baseline (286.57 W·m^−1^·K^−1^). The 700 °C-annealed coating (L_B_) raises TC to 532.93 W·m^−1^·K^−1^—an 86% increase—while the 1100 °C coating (L_D_) yields 437.98 W·m^−1^·K^−1^ (a 53% increase). Optimum performance is achieved at 900 °C (L_C_), delivering 605.27 W·m^−1^·K^−1^, which corresponds to a 111% increase (211% of the uncoated value) and the highest TC reported for Cr-based Cu/diamond composites under comparable hot-pressing conditions. These gains far exceed those obtained with molten salt Cr-carbide layers (431 W·m^−1^·K^−1^) or V- and W-based coatings (469 and 550 W·m^−1^·K^−1^, respectively), highlighting the superiority of the present annealing-tuned approach in balancing cost, uniformity, and performance.

The experimental results align closely with theoretical predictions. Diffusion mismatch model (DMM) calculations demonstrate that the partial conversion of Cr to Cr_3_C_2_ markedly improves phonon spectral overlap and interfacial adhesion energy while minimizing the acoustic-impedance mismatch between Cu and diamond. For the L_C_ interlayer (≈300 nm thick, 77.1 wt.% Cr_3_C_2_, Ra = 33.3 nm, and >98% coverage), the interfacial thermal conductance reaches 13.56 MW·m^−2^·K^−1^—1327% higher than the unmodified Cu/diamond interface—exactly as predicted by the model when an intermediate carbide layer of optimal thickness and roughness is present. In contrast, the thinner, discontinuous L_B_ coating provides insufficient bridging, while the excessively thick and coarsened L_D_ coating increases interfacial thermal resistance, confirming that both under- and over-carburization degrade phonon transport. Infrared thermography further corroborates the enhanced heat-dissipation capability: the L_C_ composite reaches 100 °C in only 12 s, 8 s faster than pure copper, directly reflecting the reduced interfacial resistance that is engineered at 900 °C.

By integrating experimentation, microstructural statistics, and multiscale simulations, this study supplies a clear processing–structure–property roadmap for Cr/Cr_3_C_2_ interlayers and demonstrates that controlled annealing at 900 °C yields the most favorable balance of bonding strength, phonon matching, and minimal thermal resistance. The resulting high-TC Cu/diamond composites (605.27 W·m^−1^·K^−1^) are, therefore, highly promising for advanced thermal-management applications in high-power-density electronics.

## Figures and Tables

**Table 1:** Phase content and thickness of the coating layer on diamond particles under different annealing conditions.

Samples	Thickness (nm)	Phase Content (wt.%)	Phase Thickness (nm)
700 °C for 2 h	194 ± 32	57.5 Cr_3_C_2_ and 42.5 Cr	115 ± 19 nm Cr_3_C_2_ and 79 ± 13 nm Cr
900 °C for 2 h	368 ± 51	77.1 Cr_3_C_2_ and 23.9 Cr	288 ± 56 nm Cr_3_C_2_ and 80 ± 5 nm Cr
1100 °C for 2 h	746 ± 94	87.2 Cr_3_C_2_ and 12.8 Cr	649 ± 83 nm Cr_3_C_2_ and 97 ± 11 nm Cr

**Table 3:** Densities, specific heat capacities, thermal diffusivities, and thermal conductivity of composites that have been reinforced by chromium-coated diamond particles after different annealing processes.

Samples	ρ (g∙cm^−3^)	$C_{p}$ (J∙g^−1^∙K^−1^)	α (mm^2^∙s^−1^)	λ (W∙m^−1^∙K^−1^)
Uncoated	5.21 ± 0.16	0.443 ± 0.08	124.16 ± 3.72	286.57 ± 8.59
700 °C for 2 h	5.46 ± 0.17	0.446 ± 0.04	218.85 ± 6.57	532.93 ± 15.99
900 °C for 2 h	5.51 ± 0.13	0.457 ± 0.05	240.37 ± 7.21	605.27 ± 18.16
1100 °C for 2 h	5.38 ± 0.15	0.449 ± 0.07	181.31 ± 5.44	437.98 ± 13.14

**Table 2:** The texture coefficients of the main crystal planes of the coating layer on diamond particles under different annealing conditions.

Samples	Cr (110)	Cr (200)	Cr_3_C_2_ (121)	Cr_3_C_2_ (230)	Cr_3_C_2_ (150)	Cr_3_C_2_ (310)
700 °C for 2 h	1.05	1.49	0.34	0.28	1.94	0.90
900 °C for 2 h	0.95	1.22	0.46	0.38	2.10	0.89
1100 °C for 2 h	1.09	0.70	0.41	0.48	2.26	1.06

**Table 4:** Parameters for the interfacial thermal conductance calculation of copper/diamond composites.

Material	*ρ* (g∙cm^−3^)	$C_{p}$ (J∙g^−1^∙K^−1^)	λ (W∙m^−1^∙K^−1^)	ν (m∙s^−1^)	Z (×10^6^ Kg∙m^−2^∙s^−1^)
Copper	8.96	385	386	2970	26.61
Diamond	3.52	510	1500	12,704	44.72
Cr	7.19	450	90	4637	33.34
Cr_3_C_2_	6.68	460	19	5493	36.69

**Table 5:** Interfacial thermal conductance and interfacial thermal resistance, as calculated by the DMM.

Interface	h (MW·m^−2^K^−1^)	R × 10^−8^ (m^2^K·W^−1^)
Cu/diamond	100.69	0.99
diamond/Cr	399.51	0.25
diamond/Cr_3_C_2_	689.72	0.16
Cr_3_C_2_/Cr	1472.75	0.06
Cu/Cr	645.77	0.15
Cu/Cr_3_C_2_	461.07	0.22

**Table 6:** Interfacial thermal conductance and interfacial thermal resistance, as calculated by the H–J model.

Interface	h (MW·m^−2^K^−1^)	R × 10^−8^(m^2^K·W^−1^)
Cu/diamond	0.95	105.26
Cu/115 ± 19 nm Cr_3_C_2_–79 ± 13 nm Cr/diamond	6.73	14.86
Cu/288 ± 56 nm Cr_3_C_2_–80 ± 5 nm Cr/diamond	13.56	7.37
Cu/649 ± 83 nm Cr_3_C_2_–97 ± 11 nm Cr/diamond	3.27	30.58

## Data Availability

The original contributions presented in this study are included in the article. Further inquiries can be directed to the corresponding author.
